# Continuous Intraarterial Nimodipine Infusion for the Treatment of Delayed Cerebral Ischemia After Aneurysmal Subarachnoid Hemorrhage: A Retrospective, Single-Center Cohort Trial

**DOI:** 10.3389/fneur.2022.829938

**Published:** 2022-03-15

**Authors:** Andreas Kramer, Moritz Selbach, Thomas Kerz, Axel Neulen, Marc A. Brockmann, Florian Ringel, Carolin Brockmann

**Affiliations:** ^1^Department of Neurosurgery, University Medical Center Mainz, Johannes Gutenberg University, Mainz, Germany; ^2^Department of Neuroradiology, University Medical Center Mainz, Johannes Gutenberg University, Mainz, Germany

**Keywords:** continuous intraarterial nimodipine infusion, aneurysmal subarachnoid hemorrhage, delayed cerebral ischemia, vasospasm, infarction, clinical outcome

## Abstract

**Background:**

Delayed cerebral ischemia (DCI) occurs after aneurysmal subarachnoid hemorrhage (aSAH). Continuous intraarterial nimodipine infusion (CIAN) is a promising approach in patients with intracranial large vessel vasospasm (LVV). The objective of this retrospective single-center cohort study was to evaluate the outcome in aSAH-patients treated with CIAN.

**Methods:**

CIAN was initiated and ended based on the clinical evaluation and transcranial Doppler (TCD), CT-angiography, CT-perfusion (PCT), and digital subtraction angiography (DSA). Nimodipine (0.5–2.0 mg/h) was administered continuously through microcatheters placed in the extracranial internal carotid and/or vertebral artery. Primary outcome measures were Glasgow Outcome Scale (GOS) at discharge and within 1 year after aSAH, and the occurrence of minor and major (<⅓ and >⅓ of LVV-affected territory) DCI-related infarctions in subsequent CT/MRI-scans. Secondary outcome measures were CIAN-associated complications.

**Results:**

A total of 17 patients underwent CIAN. Median onset of CIAN was 9 (3–13) days after aSAH, median duration was 5 (1–13) days. A favorable outcome (GOS 4–5) was achieved in 9 patients (53%) at discharge and in 13 patients within 1 year (76%). One patient died of posthemorrhagic cerebral edema. Minor cerebral infarctions occurred in five and major infarctions in three patients. One patient developed cerebral edema possibly due to CIAN. Normalization of PCT-parameters within 2 days was observed in 9/17 patients. Six patients showed clinical response and thus did not require PCT imaging.

**Conclusion:**

The favorable outcome in 76% of patients after 1 year is in line with previous studies. CIAN thus may be used to treat patients with severe therapy-refractory DCI.

## Introduction

Delayed cerebral ischemia (DCI) (1) is one of the most severe acute complications after aneurysmal subarachnoid hemorrhage (aSAH). In about 30% of patients surviving the initial hemorrhage DCI occurs, mostly between days 4 and 10 after aSAH (2, 3). Clinical deterioration such as focal neurological deficits or a decrease in consciousness is associated with DCI. It may lead to cerebral infarction with disabling neurological deficits or even death (4, 5).

Delayed cerebral ischemia is commonly accompanied by intracranial large vessel vasospasm (LVV), which can be observed using transcranial doppler sonography (TCD), CT-angiography (CTA), or digital subtraction angiography (DSA). However, the presence of an angiographic arterial spasm alone without symptomatic deterioration does not necessarily result in DCI (6, 7). The results of recent studies rather suggest a multifactorial pathophysiology of DCI, including early brain injury and thromboinflammation, microthromboembolism, impairment of microcirculation and autoregulation, as well as cortical spreading depolarizations (8–13). Nevertheless, LVV is of great clinical significance because (i) it can be detected bedside using TCD, (ii) it can be taken as a surrogate parameter for DCI in unconscious patients, triggering further diagnostics, and (iii) if associated with cerebral hypoperfusion, it can be targeted by endovascular interventions (13–15).

A variety of noninvasive treatment concepts have been evaluated, with oral nimodipine (NDP) remaining the only one with evidence of a marginal, but significant improvement in outcome (16). The classical triple-H therapy, including hemodilution, hypervolemia, and induced hypertension has not shown to be beneficial. Therefore, hemodilution and hypervolemia have been omitted in recent years, while the efficacy of the remainder of triple H therapy, induced hypertension, to prevent DCI remains unclear (17, 18). As DCI frequently remains uncontrollable with oral nimodipine alone, endovascular treatment strategies have been investigated with overall promising results (19). The most commonly used methods are short-term application of intraarterial vasodilating substances, such as nimodipine, other calcium channel blockers, or papaverine and transluminal balloon angioplasty (TBA). A downside of the pharmacological short-term treatments is the recurrence of DCI and the need for repetitive interventions (20, 21).

As a technical evolution of the above-mentioned interventions, Mayer et al. (22) described in 2008 a method for continuous infusion of nimodipine over several days using microcatheters within the cervical vessels in cases of severe refractory vasospasm. Subsequent observatory studies by other groups investigating larger patient collectives showed promising results regarding outcome, rate of complications, and side-effects (23–26).

In the underlying study, we describe our in-house experience in treating patients suffering from severe DCI with LVV with continuous intraarterial nimodipine infusion (CIAN) between May 2016 and December 2017. As proposed by a multidisciplinary research group, endpoints are the functional outcomes measured by the GOS within 1 year and cerebral infarctions in follow-up imaging (1).

## Materials and Methods

### Patient Population and Data Collection

All patients treated with CIAN for a minimum of 24 h due to severe refractory vasospasm after aSAH at our institution between May 2016 and December 2017 were included in this retrospective study. Bleeding causes other than aneurysms were excluded. Due to the retrospective nature of this study, no additional ethics committee vote was required. Routinely acquired clinical data were assessed, including all treatments associated as well as medical complications. The severity of the SAH was graded by Fisher, Hunt & Hess (H&H) and the World Federation of Neurological surgeons (WFNS) grading scale. Outcome was determined in terms of GOS at the time of discharge (GOS HD) and during a clinical follow-up visit (GOS FU) in our neurosurgical department. Additionally, radiographic data were reevaluated by an experienced and blinded neuroradiologist. Vasospasm was quantified and any new cerebral ischemic infarction was assessed. Vasospasm was graded by the magnitude of vessel narrowing in DSA (always referenced to the initial DSA) as follows: mild (<50%), moderate (50–75%), and severe (>75%). Infarctions were evaluated in routinely performed final CT scans prior to discharge and graded as suggested previously: minor (<⅓ of a vessel territory or watershed zones) and major infarction (>⅓ vessel territory) (23).

### General Patient Management

After admission noncontrast enhanced computed tomography (NECT) and CTA were performed as initial diagnostic workup. Until definite repair of the aneurysm, blood pressure was kept in a systolic range between 120 and 140 mmHg. As a secondary diagnostic tool DSA was performed within 12 h of patient admission and used as a basis for interdisciplinary decision making about a subsequent treatment. Patients underwent either microsurgical clipping or endovascular treatment (bare coil-embolization, coil-embolization with balloon- or stentremodeling, intra- or extraaneurysmal flow diversion). Within 24 h after surgical or interventional treatment, NECT and CTA were repeated as control diagnostic.

Posttherapeutic intensive care treatment was carried out according to our in-house ICU standards, which comply with the guidelines of the German Society for Neuro Intensive Care and Emergency Medicine and the German Society of Neurology. In all patients, central venous and intra-arterial catheters were established to secure drug administration and continuous blood pressure measurement. Blood glucose levels were maintained between 4.5 and 8 mmol/l (80–150 mg/dl). All patients received Dalteparin 5.000 IU subcutaneously q.d., starting on day 1 after securing the aneurysm. The flow velocities of the main cerebral arteries were controlled at least once a day by TCD.

In absence of signs of DCI, a cerebral perfusion pressure (CPP) > 70 mmHg in patients with intracranial pressure (ICP) monitoring or a mean arterial pressure (MAP) of ≥ 80 mmHg in patients without ICP monitoring was maintained. In cases of arterial hypotension, despite normovolemia, catecholamines were titrated accordingly. Nimodipine was routinely administered in a dose of 60 mg every 4 h orally or in intubated patients *via* a nasogastric tube. In cases of persistent reflux or drop in blood pressure after oral administration, it was infused intravenously.

### Diagnosis of DCI With LVV and Interventional Treatment

Awake patients were screened constantly for clinical deterioration and TCD was performed as a daily routine. In intubated patients, TCD was the main screening tool. Occurrence of new clinical deterioration, TCD flow velocities above 120 cm/s, or an increase by 50% in comparison to previous TCD examinations in one of the main cerebral arteries that was considered as surrogate parameters for DCI. When DCI was suspected, immediate further workup with NECT, CT-perfusion (PCT), and CTA was initiated to assess risk factors associated with DCI and as an evaluation tool for the degree of vessel narrowing and assessment of cerebral infarction and hemodynamic parameters (cerebral blood flow and volume, mean transit time, and time to peak). Interhemispheric asymmetry in mean transit time (MTT) by at least 1 s, vessel narrowing by > 50% in comparison to initial imaging, or proximal or distal diameter of the same vessel or vessel irregularities was rated as radiographic signs of DCI. In those cases, DSA was performed to confirm LVV and to optionally commence CIAN.

Continuous intraarterial nimodipine infusion was initiated if DSA showed severe (> 75% narrowing of vessel diameter compared to initial DSA) or diffuse LVV in clinically deteriorating patients or if in sedated or comatose patients additional perfusion deficits in PCT were present. It was also initiated in case of persisting clinical symptoms despite short-term IAN in patients with moderate LVV. In the absence of severe LVV and perfusion deficits in PCT, MAP was maintained above 100 mmHg by infusing norepinephrine and crystalloid solutions. Induced hypertension was continued during CIAN, although we refrained from using excessive doses of vasopressors as nimodipine-associated cerebral vasodilation normally improves cerebral perfusion, thereby gradually reducing the need for induced hypertension.

For CIAN, the common femoral artery was routinely used to obtain vascular access with a 6F introducer sheath. One or 2 microcatheters were placed in the internal carotid artery (ICA) or vertebral artery (VA) depending on the site of LVV or clinically suspected DCI. Nimodipine (Nimotop^®^ S, 10 mg/50 ml, Bayer Vital GmbH GB Pharma, Germany) was initially administered at a rate of 2 mg/h (10 ml/h) in combination with 10 ml/h of Heparin (1 IU/ml), resulting in an infusion rate of 20 ml/h to prevent catheter obstruction or thrombus apposition. On dependency of the clinical and radiographic course of the individual patient, the nimodipine administration rate was then reduced successively. Intravenous platelet inhibition with Tirofiban (Aggrastat^®^, MSD) in a body-weight adapted dose (initial infusion rate of 0.4 μg/kg/min for 30 min followed by a maintenance dose of 0.1 μg/kg/min) was conducted to prevent thromboembolic complications.

During CIAN intensive care including induced hypertension was continued as described. In sedated patients with additional ICP monitoring, blood pressure was adjusted according to CPP. In case of continuous or new signs of DCI such as neurological symptoms or elevated flow velocity in TCD, NECT, CTA, and PCT were repeated. In patients who were awake CIAN was ended when neurological symptoms resolved and flow velocities in TCD normalized. In intubated patients CIAN was ended when PCT or DSA and flow velocities normalized. In any case, the infusion rate of nimodipine was reduced gradually over 1–3 days to prevent a rebound of LVV and/or symptoms.

### Study Endpoints

Two parameters were evaluated as primary endpoints: 1) Clinical outcome according to the Glasgow Outcome Scale at discharge (GOS HD) and at follow-up within 12 months (GOS FU) after the subarachnoid hemorrhage and 2) Presence of cerebral infarction assessed on the most recent CT scan prior to discharge with a rating of infarction as described above. As a secondary endpoint interventional treatment associated complications such as catheter tip thrombosis, catheter occlusion, and vessel dissection were evaluated.

### Statistical Analyses

For statistical analysis of outcome parameters R version 4.1.2 was used with Spearman's rank correlation for ordinal variables, Phi coefficient for binary variables (Chi-Square for significance), and Mann-Whitney U test in case of a combination of ordinal and binary variables. Statistical significance was defined as a *p* ≤ 0.05.

## Results

### Patient Baseline Characteristics

During the investigation period, we treated a total of 54 patients with aSAH in our department. Of these, 17 patients (31.5%; 5 male, 12 female) suffered from refractory DCI and thus were treated with CIAN. The patients' age ranged from 38 to 72 years with a median age of 54. Nine out of the 17 patients were graded Fisher 4, the remaining 8/17 patients were graded Fisher 3. H&H as well as WFNS grading ranged from 1 to 5 (median 2). At admission, 8/17 patients showed a neurological deficit, and the Glasgow-Coma-Scale (GCS) ranged from 3 to 15 (median 14).

Delayed cerebral ischemia with associated angiographic LVV occurred 9 days (median) after hemorrhage (range: 3–13 days). Relevant LVV was observed in DSA most frequently in the middle cerebral artery (MCA) (16/17) and the anterior cerebral artery (ACA) (13/17), and in only three cases in the ICA. Most patients (13/17) presented with LVV of multiple cerebral arteries at the same time point, three patients showed LVV of the MCA, and one patient of the ACA only. Table 1 provides patients' demographics, SAH classification, treatment modalities, risk factors and outcome.

**Table 1:** Demographic data, SAH classification and risk factors, and outcome of CIAN patients.

**Patient ID**	**Age**	**Gender**	**Fisher**	**H&H**	**IVH**	**ICH**	**Aneurysm**	**Treatment**	**Sight of VS**	**IAN**	**TBA**	**Risk factors**	**Infarction**	**GOS HD**	**GOS FU**
1	44	w	3	2	n	n	MCA l	Coiling	MCA l	y	y	-	N	5	5
2	51	m	3	1	n	n	MCA r	Clipping	MCA r	n	y	-	N	5	5
3	60	w	4	5	n	n	ACA l	Coiling	ACA r, MCA r	y	y	-	Major	1	-
4	52	w	4	5	n	n	VA l	Flow diverting stent	ACI r/l, MCA r/l, ACA r/l, PCA r/l, BA	y	y	aHT	Major	2	3
5	72	w	4	5	y	y	AComA	Coiling	MCA li, BA, PCA r/l	n	n	aHT	N	2	-
6	68	w	4	1	y	n	ACI r	Coiling	ACA r/l, MCA r/l	n	n	-	N	5	5
7	38	m	4	2	y	y	AComA	Clipping	ACI r/l, ACA r/l, MCA r/l	y	n	-	N	2	5
8	57	m	4	5	n	n	BA, MCA r	Coiling, Clipping	ACA r, MCA r	n	y	Nicotine aHT	Minor	4	5
9	61	w	3	5	n	n	AComA	Coiling	MCA r, ACA r	n	n	Nicotine	Minor	3	4
10	45	w	4	5	y	n	PComA l	Coiling	ACA r, MCA l	n	n	Nicotine aHT	Minor	3	5
11	39	m	3	3	n	n	MCA l, ACI l	WEB	MCA l, ACA l	n	n	Nicotine	Minor	3	4
12	53	w	4	2	y	y	AComA	Coiling	ACI r/l, MCA r/l, ACA r/l	n	n	-	N	5	5
13	43	w	3	2	n	n	MCA r,	Clipping	ACI r, MCA r	n	n	-	N	5	5
							AcomA								
14	50	m	4	1	y	y	ACI l	Coiling	MCA l, ACA l, ACI l	n	n	aHT	N	5	5
15	54	w	3	1	n	n	MCA r	Clipping	MCA r, ACA r, ACI r	n	n	aHT	N	5	5
16	51	w	3	2	n	n	AcomA	Coiling	ACA r/l	y	n	aHT	Minor	5	-
17	61	w	3	2	n	n	MCA r	Clipping	MCA r, ACA r	n	n	-	Major	3	-

*H&H, Hunt & Hess; GCS, Glascow Coma Scale; IVH, intraventricular hemorrhage; ICH, intracerebral hemorrhage; WEB, Woven EndoBridge (intraaneurysmal Flow-Diverter); FD, extraaneurysmal Flow-Diverter; MCA, middle cerebral artery; ACA, anterior cerebral artery; VA, vertebral artery; AcomA, anterior communicating artery; PcomA, posterior communicating artery; PCA, posterior cerebral artery; r, right; l, left; IAN, intra-arterial nimodioine infusion; TBA, transluminal balloon angioplasty; aHT, arterial hypertension. Minor infarctions were considered infarctions affect less than one third of a vascular territory. Major infarcts were considered infarctions affecting more than one third of a vascular territory. GOS HD, GOS at hospital discharge; GOS FU, GOS within 1 year*.

### CIAN Treatment Characteristics

The median treatment duration of CIAN was 5 days (range 1–13). A single microcatheter was placed in the ICA in 11/17 cases and one catheter was placed into the VA. In the remaining patients (5/17) catheters were placed bilaterally into the ICA. Before commencing CIAN, TBA was aborted in 4/17 patients, and a short-term intraarterial nimodipine infusion (IAN/30 min) prior to CIAN was carried out in 5/17 patients with insufficient results.

### Complications and Side Effects

General complications were hospital-acquired pneumonia (HAP) in four cases, in two cases with prolonged weaning and need for a tracheostomy, as well as prolonged weaning without infection in one case. Additionally, five cases of urinary tract infections, one meningitis, one acute respiratory distress syndrome on the basis of a fulminant pulmonary embolism, one tension pneumothorax, one cholangitis, and one temporary cardiomyopathy of unknown origin occurred. One patient had an epileptic seizure and one patient died due to a malignant brain edema after multiple brain infarctions.

Complications related to CIAN were pressure elevation in five microcatheters of four patients without evidence of catheter tip thrombosis and accidental catheter removal in two patients. This resulted in six catheter replacements in four patients. One patient developed a localized temporary cerebral edema as a suspected side effect of CIAN.

Other complications were one perforation of a M2 branch of the MCA during TBA prior to CIAN. In two cases dissections of left-sided ICA (C2 segments) occurred, one during unsuccessful TBA prior to CIAN and one was detected after ending CIAN. Both dissections did not result in vessel occlusion. No cases of catheter-associated ischemic events occurred.

### Treatment Response

Before initiation of CIAN, DSA showed severe vasospasm in seven patients and moderate vasospasm in nine patients. In one case microcatheters were placed directly without additional imaging of the cerebrovasculature prior to CIAN. All patients showed focal hypoperfusion in PCT. At the time point when CIAN was started 10/17 patients were awake and showed immediate clinical improvement after initiation of CIAN. Seven patients were already sedated or unconscious at CIAN onset, of which five patients showed decreasing TCD-velocities as a result of CIAN. One patient remained with increased TCD-velocities, with PCT showing residual hypoperfusion. One sedated patient was not assessable by TCD but presented with hyperperfusion of treated vessel territory in PCT 1 day after initiation of CIAN, and so the intraarterial nimodipine infusion was stopped.

In eight cases first follow-up CT imaging (NECT, CTA, and PCT) was performed on day 1 after commencing CIAN, in three cases on day 2, and in each one case on day 3, 5, and 8. In all the cases follow-up imaging showed reduced LVV and normalization or even reversal of PCT-parameters (Table 2). The remaining three patients did not undergo follow-up imaging because of a favorable clinical course during CIAN.

**Table 2:** Treatment response.

**Patient ID**	**DSA**	**CTA**	**PCT**	**Follow-up CTA/PCT**													**Duration of CIAN therapy (days)**	**Clinical improvement**
	**Vasospasm grading on day 0 (=** **start of CIAN)**			**day 1**	**day 2**	**day 3**	**day 4**	**day 5**	**day 6**	**day 7**	**day 8**	**day 9**	**day 10**	**day 11**	**day 12**	**day 13**		
1	2	2	↓								0/↔						2	y
2	n/a	2	↓														1	y
3	3	3	↓	3/↓	0/↔	0/↔	0/↔				1/↓						7	s/TCD↔
4	2	3	↓	1/↓			1/↔		0/↔			0/↔			1/↔		6	s/TCD↓
5	2	2	↓	0/↑		0/↔		0/↔									1	s/no TCD
6	2	3	↓	0/↑	0/↔		0/↔					0/↔			0/↔		3	y
7	2	2	↓	0/↑			1/↔		1/↔		0/↔						7	y
8	2	2	↓		0/↔		0/↔				0/↔				0/↔		5	s/TCD↓
9	2	2	↓		0/↑												5	y
10	3	3	↓			0/↑	0/↔										3	s/TCD↓
11	3	3	↓	0/↑		3/↓	0/↔		1/↔		2/↓		1/↔			0/↔	13	s/TCD↓
12	3	3	↓	0/↔	0/↔		1/↔		1/↔					1/↔			4	s/TCD↓
13	2	2	↓														3	y
14	3	2	↓					1/↑	1/↓								5	y
15	3	3	↓														3	y
16	3	2	↓		2/↓		2/↔		0/↔		0/↔		0/↔	0/↔			10	y
17	2	3	↓	2/↑		0/↔		2/↓	0/↔								9	y

*Vasospasm in DSA and CTA was graded as 0 = no relevant vasospasm, 1 = mild vasospasm, 2 = moderate vasospasm, 3 = severe vasospasm. ↓, ↔, and ↑ indicate hypo-, normo-, or hyperperfusion in PCT. Gray-shaded fields indicate days with CIAN therapy. Clinical reaction: y = yes, s = sedated, TCD↔ = flow velocity in TCD unchanged, TCD↓ = flow velocity in TCD reduced*.

Three patients showed recurrent LVV and hypoperfusion in PCT during CIAN, which in all cases were associated with new cerebral infarction at follow-up imaging. There were recurrent mild LVV without a perfusion deficit during CIAN in five patients. Table 2 gives an overview of the patient's course prior to and during CIAN.

### Outcome

At discharge about half of the patients (8/17) showed DCI-related cerebral infarcts which could not be explained by other causes. Five were graded as minor, hereof four in ACA- and one in MCA-territory, and three as major, hereof two in the ACA- and one in the MCA-territory. Infarcts were preceded by LVV of the supplying vessel in all cases.

The functional outcome was graded by the GOS. A favorable outcome was defined as a GOS of 4–5. At hospital discharge, GOS (GOS HD) of 9/17 patients showed a favorable outcome. Of the 13/17 cases with available follow-up data within 1 year (median 12 months, range 6–12 months) GOS (GOS FU) of four patients improved from unfavorable to favorable outcome. Assuming an unaltered GOS of the four patients without follow-up data within 1 year resulted in a favorable outcome in 13/17 patients.

Patients/treatment characteristics and outcome parameters (GOS HD, GOS FU, and infarctions at discharge) were correlated using Spearman's rank correlation, phi coefficients, or Mann–Whitney U Test depending on the type of data used (Table 3).

**Table 3:** Statistical analyses of outcome parameters.

**Parameter**	**GOS HD**		**GOS FU**		**Infarcts**	
	***r***	***p*-value**	***r***	***p*-value**	***r***	***p*-value**
H&H (1–5)	**−0.753**	**<0.001**	**−0.600**	**0.011**	**0.650**	<0.001
Fisher (1–4)	−0.385	0.127	−0.070	0.788	−0.054	<0.001
Age (y)	−0.166	0.524	−0.463	0.061	0.129	<0.001
GCS (1–15)	**0.808**	**<0.001**	**0.552**	**0.022**	**−0.534**	**<0.001**
Gender (female)	−0.014	<0.001	−0.083	0.006	0.091	1.000
IVH (y/n)	−0.026	<0.001	0,413	0.006	−0.450	0.130
ICH (y/n)	−0.088	<0.001	0.286	0.005	**−0.523**	**0.081**
Neurological deficit (y/n)	**−0.834**	**<0.001**	**−0.563**	**0.006**	**0.528**	**0.058**
Start CIAN (d)	−0.075	0.775	−0.240	0.354	0.332	<0.001
Duration CIAN (d)	−0.373	0.141	−0.318	0.214	**0.635**	<0.001
EVD (y/n)	−0.247	<0.001	−0.349	0.006	0.203	0.620
Treatment (EVT)	0.0252	<0.001	−0.081	0.006	0,071	1.000
IAN (y/n)	−0.301	<0.001	−0.087	0.006	0.290	0.335
TBA (y/n)	−0.136	<0.001	−0.087	0.006	0.167	0.618
Infarcts (y/n)	−0.485	<0.001	**−0.660**	**0.006**	–	–
Hypertension (y/n)	−0.025	<0.001	0.000	0.006	0.169	0.636
Nicotine (y/n)	−0.219	<0.001	−0.035	0.006	**0.588**	**0.030**
Preinterventional	0.130	0.619	0.100	0.702	0.164	0.317
VS-Grading, CTA (1–3)						
Highest VS-grading	−0.268	0.299	−0.464	0.060	0.420	0.553
during CIAN, CTA (1–3)						
Hypoperfusion	−0.395	<0.001	**−0.779**	**−0.004**	**0.685**	**0.010**
during CIAN (y/n)						

*Spearman's rank correlation for ordinal variables, Phi Coefficient for binary variables (Chi-Square for significance), and Mann–Whitney U-test in case of a combination of ordinal and binary variables. Statistical significance was defined as a p ≤ 0.05. GOS HD, Glasgow outcome score at hospital discharge; GOS FU, Glasgow outcome score at follow-up within 1 year; infarcts, radiological evidence of DCI associated infarcts. At least moderate correlations are highlighted in bold (r > 0.5)*.

The GOS HD is highly negative and significantly correlated with the presence of neurological deficits at admission (*r* = −0.834, *p* < 0.001) and the severity of aSAH according to H&H (*r* = −0.753, *p* < 0.001) and highly positive and significantly with GCS at admission (*r* = 0.808, *p* < 0.001).

The GOS FU is moderately negative and significantly correlated with the severity of aSAH according to H&H (*r* = −0.600, *p* = 0.011), moderately positive and significantly correlated with GCS (*r* = 0.552, *p* = 0.022), and moderately negative and significantly correlated with the occurrence of neurological deficits at admission (*r* = −0.563, *p* = 0.006).

Occurrence of DCI-associated infarcts is moderately positive and significantly correlated with the severity of aSAH according to H&H (*r* = 0.650, *p* < 0.001), moderately negative and significantly correlated with GCS at admission (*r* = −0.543, *p* < 0.001), moderately negative and nonsignificantly correlated with the occurrence of ICH (*r* = −0.523, *p* = 0.081), and moderately positive and non-significantly correlated with the presence of neurological deficits at admission (*r* = 0.528, *p* = 0.058).

In addition, occurrence of DCI-associated infarcts is moderately positive and significantly correlated with the risk factor nicotine consumption (*r* = 0.588, *p* = 0.030), moderately positive and significantly correlated with the duration of CIAN (*r* = 0.656, *p* = 0.004), and moderately positive and significantly correlated with the occurrence of hypoperfusion during CIAN (*r* = 0.685, *p* = 0.010).

### Illustrative Case

**Figure 1:**
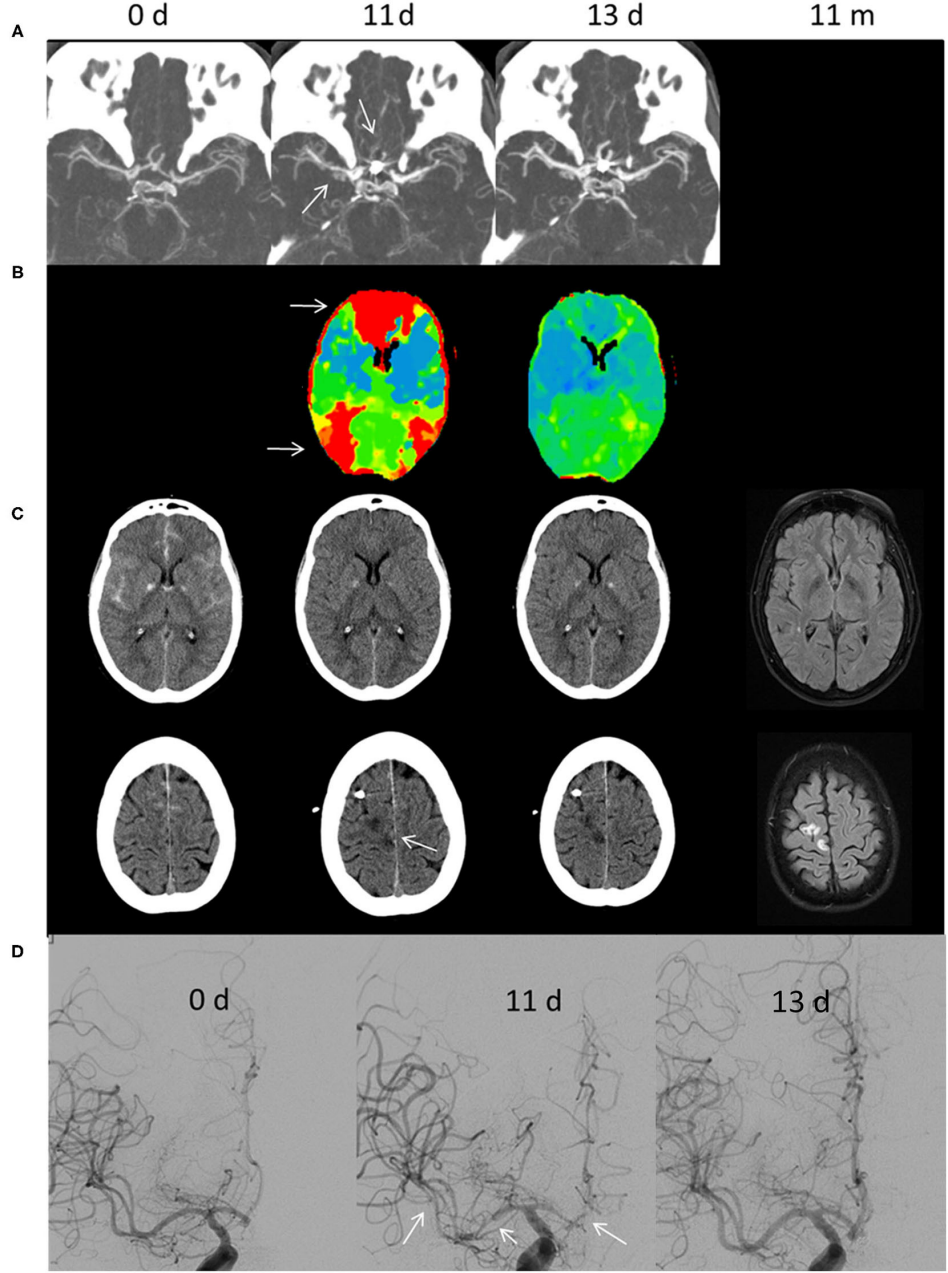
Illustrative case of CIAN therapy. **(A)** CT angiography, **(B)** perfusion CT, **(C)** cranial CT and T2-weighted fluid-attenuated inversion recovery, and **(D)** digital subtraction angiography. The time is given in days (d) and months (m) after aSAH. The initial cranial CT shows aSAH Fisher Grade III due to rupture of an AcomA-aneurysm, which was subsequently treated by coil embolization. On day 11, the MTT (CBV normal, not shown) was prolonged predominantly in the ACA and distal MCA/watershed territories (white arrows in **B**). In addition, a small sub-/cortical infarction within the right ACA territory was diagnosed (arrow in **C**). DSA showed severe large vessel vasospasm of the right MCA and ACA segments (arrows). CIAN was commenced immediately. Two days later (day 13) MTT in the ACA territory normalized and no new infarctions could be diagnosed in CT. Likewise, vessel diameter normalized as shown in DSA. In a follow-up MRI 11 months later no new infarctions were detectable.

A 63-year-old female developed reduced vigilance (H&H 5, GCS 5) after initial thunderclap headache and was intubated by the emergency physician. Initial NECT and CTA showed subarachnoid hemorrhage Fisher 3 without intraventricular hemorrhage and an AcomA-aneurysm which was subsequently treated by coil-embolization (Figure 1). An external ventricular drainage was placed after signs of hydrocephalus were present.

After reduction of sedation, the patient could be extubated on the 3rd day post-SAH. At this time, no focal neurological deficits were present. Increased TCD values on the 5th and 9th day triggered CTA and PCT with no apparent perfusion deficit, whereas mild to moderate LVV was present.

Conservative therapy was initiated with normovolemic hypertension and oral nimodipine (3 × 60 g).

On day 11, DCI was suspected as increased TCD values were accompanied by a left-sided hemiparesis. CTA showed severe LVV of the right ACA and MCA, and PCT showed an increase of MTT predominantly in the right ACA-territory and to a lesser extent in the right MCA-territory. Additionally, there was a small acute cortical/subcortical infarct in the right upper ACA-territory (superior frontal gyrus).

Digital subtraction angiography was performed and confirmed severe vasospasm (> 75% vessel narrowing) in the middle and proximal M1-segments; several M2-segments; and A1-, A2-, and A3-segments. A microcatheter was placed in the extracranial right ICA. CIAN was carried out according to our protocol with 2 mg NDP/h, a flow rate of 10 ml/h, and tirofiban, dosed according to the patient's weight.

During the first hours of treatment, hemiparesis subsided and TCD-values normalized. PCT and CTA on day 13 showed dilation of intracranial vessels exceeding baseline values at admission and normalization of cerebral perfusion. Five days later CIAN was stopped after gradually decreasing the infusion rate and the patient's clinical status remained unchanged. No adverse events occurred; norepinephrine was administered as circulatory support.

The patient was discharged at day 23 without any focal neurological deficit. Limitations in concentration and cognitive functions were present, thus the patient was scored with a GOS of 3. Follow-up examination after rehabilitation treatment showed an improvement resulting in a favorable GOS of 4 after 3 months and 1 year, respectively.

## Discussion

We retrospectively analyzed a cohort of 17 patients treated with CIAN for severe therapy refractory vasospasm after aSAH. With regard to a total of 54 patients treated for aSAH in the inclusion time window, our study cohort's proportion matches those of published data (27). The purpose of this study was to evaluate the safety of the CIAN technique and the clinical outcome of the treated patients. First, we did not observe severe adverse events attributable to CIAN during this period. Only in one patient, we observed a vasogenic cerebral edema which we attributed to treatment with CIAN, and which resolved after immediate termination of CIAN therapy. Another patient presented with an ICA dissection, the exact pathomechanism of which remained unclear (possibly during placement of microcatheter or even unrecognized during an earlier intervention). No vessel occlusions occurred during treatment with CIAN. Complications reported by other groups included thrombus suspected wall alterations in CTA (which did not cause any embolic infarctions) (26), four cases of catheter tip thrombosis, and one case of ICA dissection during CIAN using much larger and stiffer 4F catheters (28), as well as catheter occlusion in three cases. Since in our study we have been using quite soft 1.7 F microcatheters (normally used for coil embolization), we observed no case of catheter tip thrombosis during CIAN, which also might be due to the effective (or even aggressive) platelet inhibition using tirofiban. Catheter occlusion was also observed in our study in four cases, although the exact reason for the increasing infusion pressure cannot be easily found [thrombotic occlusion or precipitation of nimodipine, as reported by others (23)].

As mentioned above, anticoagulation or platelet inhibition plays an important role when foreign materials (like catheters) are left within the vessel lumen. Mainly heparin [Ott et al. (26) Heparin 10,000 IU/24 h; Bele et al. (23): Heparin guided by a PTT of 50–60 s] and tirofiban are currently being used to prevent thromboembolic events in this specific setting. Since platelet activation, adhesion, and aggregation play a significant role in thromboembolic complications during endovascular procedures (29, 30), and GPIIb/IIIa-receptor inhibitors (such as tirofiban) have been shown to be effective and safe in prophylaxis and rescue therapy in neurovascular interventions (31), we decided to use platelet inhibition. As microthrombosis has also been discussed to contribute to DCI, platelet inhibition or anticoagulation might even have a positive side effect. Most important, no severe bleeding complications despite antiplatelet therapy and ventricular drains in place during CIAN occurred in this cohort.

Types and rates of intraarterially infused medication vary. Although minirin and papaverin have also been used to treat vasospasm, we applied nimodipine 2 mg/h, which is a commonly used dosing. Other groups used slightly lower doses ranging from 0.4 to 1.6 mg/h (23–26, 32). Kieninger et al. (33) reported in 2019 that venous plasma levels of intraarterially administered nimodipine were lower than those of enterally given 60 mg nimodipine, if the application rate was below 1 mg/h. Nimodipine is well-known to undergo extensive first-pass metabolism in the liver and gut wall, which to some extent might also contribute to a reduced efficacy of systemically applied nimodipine. Regarding plasma levels of nimodipine, pharmacokinetic modeling of CIAN-therapy for SAH-related DCI using a two-compartment pharmacokinetic model was assessed earlier (34). This study showed that CIAN allows rapid initiation of high dose therapy, with about 85% of the maximal concentration of nimodipine being achieved within the first minute of intraarterial infusion, independent of the infusion dose (calculated for infusion rates of 1 and 2 mg/h). Furthermore, at IA infusion rates of 1 and 2 mg/h, the calculated intraarterial concentration of nimodipine reached 100 and 200 ng/ml, which was significantly higher compared to the calculated steady-state concentration of 29 ng/ml during continuous IV infusion of nimodipine at 2 mg/h, which might very well explain the increased efficacy of the CIAN technique compared to IV infusion or oral administration.

In Kieninger et al. (25) also reported that higher rates of vasopressors during CIAN compared to orally administered nimodipine were required to avoid blood pressure drops and therefore insufficient CPP. Furthermore, generally high doses of sedatives to maintain a RASS-5 score during CIAN can surely also contribute to the higher rate of vasopressors doses. Although blood pressure drops induced by nimodipine might need to be counteracted by higher doses of vasopressors, it remains questionable as to what degree hypertension needs to be maintained to avoid insufficient CPP in patients with good response to intraarterially administered nimodipine. In contrast to other reports (25, 32, 35), we did not observe relevant hemodynamic side effects requiring a discontinuation of CIAN therapy. Likewise in this cohort, we did not observe cases of cardiac arrest as reported by Kieninger et al. (25) (one case of tachycardia and one case of asystolia), although we experienced comparable complications earlier at other sites. With four cases of HAP and five urinary tract infections, general complications seem to be in an appropriate range for an ICU treatment (36). Kienigner et al. (25) however, reported a higher rate of infectious complications during CIAN, which they interpreted in context with a prolonged sedative therapy and immobilization in supine position and prolonged weaning. Whether CIAN in awake and potentially uncooperative patients may yield a higher risk of complications, such as catheter disconnection or dislocation, remains unclear. The risk of ICA dissection using the extremely small and soft microcatheters from our experience can be neglected unless dissection occurs during catheter placement. In our patient cohort, we experienced no severe adverse events attributable to awake patients' mobility.

Diagnosis of DCI was made after assessing clinical (if feasible) and (in any case) radiological parameters, including TCD, CCT, CTA, and PCT. Angiography was finally used to confirm severe LVV and to ultimately indicate treatment with CIAN. This corresponds to the treatment protocols of most other publications, although Kienienger et al. (25) and Bele et al. (23) used CTA only without PCT to make a treatment decision. In our study, classification of vasospasm led to subtle differences between CTA and DSA regarding moderate vs. severe vasospasm in five cases, which may be due to insufficient contrast in some CTA examinations and borderline cases (with the degree of vasospasm being close to the border between moderate and severe). These differences, however, do not seem to be highly relevant as even grading of vasospasm differs between studies. For example, Musahl et al. (32) used a grading comparable to our study, whereas Hockel et al. (24) interpreted a vessel narrowing of > 60% as severe vasospasm.

In other publications, multimodal neuromonitoring (MNM), especially the measurement of oxygen partial pressure in the brain (PtiO2), pyruvate metabolism, and rCBF, were used for treatment decision and monitoring. PtiO2 values have been reported to correlate with vasospasm as documented by concomitant radiographic examinations (23, 37). Therefore, implementation of PtiO2 measurement could be considered when obligatory sedation or impaired vigilance are impeding the patients' continuous clinical evaluation. Other authors also used DSA (26, 35) or PCT (28) in predefined time intervals after initiation of CIAN as monitoring tools. For example, Kieninger et al. (25) removed the microcatheters if the rCBF was above 20 ml/100 g/min and PtiO2 was above 15 mmHg 24h after termination of CIAN. Advantages of intraparenchymal measurements include continuous and quantitative monitoring values, whereas invasiveness and limited spatial tissue coverage are disadvantages. Nonetheless, these monitoring techniques might be of interest when developing general guidelines for CIAN management.

Logistic efforts and transportation associated complications and repetitive exposure to radiation and contrast agent have to be considered as disadvantages of these imaging modalities. Furthermore, once microcatheters are in place, repetitive diagnostic DSA normally is not feasible through microcatheters. Regarding radiation dose issues, efforts are being made to reduce radiation dose by adapting PCT protocols (38–41) and, more recently, by implementation of artificial intelligence-aided image reconstruction, which would improve the quality of ultra-low-dose CT imaging.

Continuous intraarterial nimodipine infusion was commenced in our cohort 9 days (median) after SAH, which lies within the typical interval for occurrence of DCI (3). The median duration of CIAN treatment in our cohort with 5 days was comparable to reports from other groups (20, 23–26), although recently published studies reported significantly longer median CIAN treatment durations of up to 10–12.5 days (25). The observed prolongation of CIAN treatment in these most recent studies might reflect the positive experience (i.e., encouraging clinical results and low complication rates) with this technique.

Consistent with the results of Musahl et al. (32), all of our awake patients showed clinical improvement after CIAN initiation within 24 h. Other study groups sedated patients during CIAN, and so the clinical evaluation of these subgroups is lacking (24, 25). The outcome of our cohort is comparable to the outcome reported in the literature: Bele et al. (23) reported 76% and Kieninger et al. (25) 61% favorable outcome after 6 months. In these studies, GOS improved over time between discharge and follow-up examination, which has been also reported in other studies (42, 43). Regarding radiographic outcome, Bele et al. (23) reported a comparable percentage of DCI-associated infarctions (42.9% overall; 10% major, 90% minor infarction) and found this to be significantly less than in their historical control group (75% overall). In a 2017 published meta-analysis by Boulois et al. (44) conservative and endovascular treatment of DCI were compared. In a subgroup analysis of seven RCTs investigating patients suffering from severe refractory vasospasm, conservatively treated patients in 50% had an unfavorable outcome, whereas patients undergoing interventional endovascular treatment in only 34% of cases showed an unfavorable outcome. This data matches well with our findings; CIAN patients of our cohort recovered to a relatively high percentage of favorable outcome, especially within 1 year after discharge (76%).

Major limitations of the presented study are its retrospective single center design, the lack of a control group, and a small sample size. As a potential selection bias, we have to acknowledge that, compared to other groups, our protocol accords greater weight to cerebral hypoperfusion measured by PCT, which may lead to a comparably higher rate of CIAN treatments in our cohort. Concerning the rate of treatment-related infarctions additional MRI imaging prior to hospital discharge may have resulted in higher numbers. This, however, would have resulted in a greater logistical expense, and finally the pathogenesis of smaller, subacute ischemic lesions (postoperative, postinterventional, CIAN-related, and DCI-related) might not have been correctly identified. By classifying infarctions depending on their volume into major and minor infarction, the anatomical localization in eloquent areas with comparably higher impact on patients' functional status has not been taken into account. Since the management protocol of our clinic did not intend routine visits after more than 1 year, long-term follow-up data are lacking. This again might lead to an overrepresentation of poor outcomes in our cohort, as other long-term outcome studies showed a steady increase of outcome over time (42, 43). Prospective RCTs will be required to clearly confirm these mostly positive results of single center cohort studies and to identify precise inclusion and monitoring criteria for CIAN-eligible patients. Another critical point is that few patients in our cohort underwent TBA (in two cases with a complication) or short-term IAN prior to CIAN. Although this study, aimed at investigating CIAN and was not a RCT, we decided to include these patients anyway (as they underwent CIAN), as the previous procedures (TBA/IAN) for different reasons did not yield the desired results or were aborted at an early time point. It is well-known that other studies report TBA to be a safe and effective in the treatment of LVV (45, 46), and that from the few TBA cases included in this study no conclusion regarding the efficacy of TBA can be drawn. Interestingly, other devices like stentretrievers normally used for thrombectomy (47) and adjustable remodeling meshes developed to aid in aneurysm coiling (48) are currently being investigated for endovascular treatment of severe vasospasm.

Overall, we found a high percentage of CIAN-treated patients to recover to a favorable outcome; hence we can confirm CIAN to be a therapeutic option for patients severely affected by refractory vasospasm after aSAH.

## Data Availability Statement

The original contributions presented in the study are included in the article/supplementary material, further inquiries can be directed to the corresponding authors.

## Ethics Statement

Ethical review and approval was not required for the study on human participants in accordance with the local legislation and institutional requirements. Written informed consent for participation was not required for this study in accordance with the national legislation and the institutional requirements.

## Author Contributions

AK, MS, TK, and CB are accountable for data acquisition. Statistical analysis was performed by MS and AK. AK, MS, AN, MB, FR, and CB are accountable for the preparation of the manuscript and study design. Results published in this manuscript are a parts of MS doctoral thesis. All authors agree to be accountable for the content of the work.

## Conflict of Interest

The authors declare that the research was conducted in the absence of any commercial or financial relationships that could be construed as a potential conflict of interest.

## Publisher's Note

All claims expressed in this article are solely those of the authors and do not necessarily represent those of their affiliated organizations, or those of the publisher, the editors and the reviewers. Any product that may be evaluated in this article, or claim that may be made by its manufacturer, is not guaranteed or endorsed by the publisher.
